# Application of Probiotics in Foods: A Comprehensive Review of Benefits, Challenges, and Future Perspectives

**DOI:** 10.3390/foods14173088

**Published:** 2025-09-02

**Authors:** Mirjana Ž. Grujović, Teresa Semedo-Lemsaddek, Katarina G. Marković

**Affiliations:** 1 Department of Science, Institute for Information Technologies, University of Kragujevac, Jovana Cvijica bb, 34000 Kragujevac, Serbia; mirjana.grujovic@pmf.kg.ac.rs; 2 Centre for Interdisciplinary Research in Animal Health (CIISA), Faculty of Veterinary Medicine, University of Lisbon, Av. da Universidade Tecnica, 1300-477 Lisbon, Portugal; 3 Associate Laboratory for Animal and Veterinary Sciences (AL4AnimalS), 1300-477 Lisbon, Portugal; 4 BioISI-Biosystems & Integrative Sciences Institute, Faculty of Sciences, University of Lisbon, 1749-016 Lisbon, Portugal

**Keywords:** probiotics, functional foods, fermented foods, food matrices, probiotic delivery systems

## Abstract

The incorporation of probiotics into food products has gained substantial attention, primarily due to their well-documented health benefits such as modulating gut microbiota, enhancing immune responses, and providing potential therapeutic effects. This comprehensive review discusses recent advancements in the application of probiotics in the food industry, focusing on diverse food matrices, technological and regulatory challenges, and consumer acceptance. Particular emphasis is placed on fermentation-based approaches that enhance both sensory and nutritional attributes, while acting as effective delivery systems for viable probiotics. The impact of matrices such as dairy, meat, cereals, plant-based beverages (e.g., soy or almond milk), and solid plant-derived foods (e.g., fermented vegetables) on probiotic survival, sensory properties, and product acceptability is critically examined. Understanding these interactions is crucial for the development of stable, efficacious, and consumer-oriented probiotic-enriched functional foods.

## 1. Introduction

Probiotics are live microorganisms that, when administered in adequate amounts, confer health benefits to the host [1,2]. Among these, lactic acid bacteria (LAB) and certain yeasts are the most frequently used in fermented food products. *Lactobacillus* and *Bifidobacterium* species are the predominant bacterial genera due to their long-standing safety record and proven efficacy in modulating gut microbiota, enhancing immune function, and improving digestive health [3]. *Lacticaseibacillus rhamnosus* (formerly classified as *Lactobacillus rhamnosus*), *Lactobacillus acidophilus*, *Lacticaseibacillus casei* (formerly classified as *Lactobacillus casei*), and *Bifidobacterium lactis* are particularly well-studied for their roles in dairy products such as yogurt, kefir, and fermented milk [3,4]. Additionally, *Streptococcus thermophilus* is commonly used alongside *Lactobacillus* strains in yogurt production to initiate fermentation and improve product texture and flavor [5]. Among yeasts, *Saccharomyces boulardii* stands out as a well-established probiotic, effective against gastrointestinal disorders, while *Saccharomyces cerevisiae* is widely utilized for its fermentation properties and emerging probiotic potential. Other non-conventional probiotic yeasts like *Kluyveromyces marxianus*, *Debaryomyces hansenii*, *Candida milleri*, and *Yarrowia lipolytica* are gaining attention for their functional contributions to flavor, texture, and enzyme production in dairy and non-dairy fermented foods [6].

**Figure 1:**
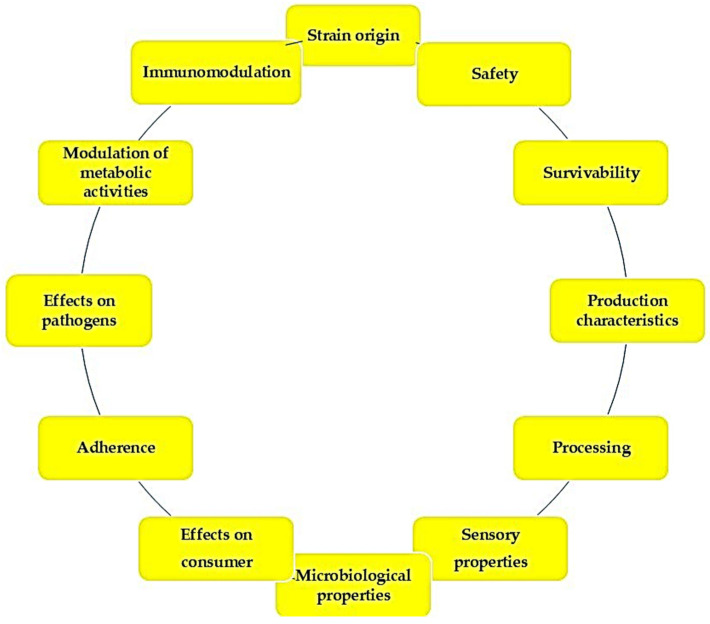
Characteristics of probiotic strains (inspired by Lau & Quek [22]).

Although there is a wide variety of probiotic strains available on the market, the search for new probiotics continues [7,8]. Various criteria have been proposed to standardize the desirable characteristics (Figure 1), helping to identify those that meet the required selection standards and exclude those that do not. Probiotics obtained from the same species are more likely to exhibit enhanced survival chances, as demonstrated by Mahmoudi et al. [9]. Safety is a significant concern, with probiotics needing to be generally regarded as safe (GRAS) in the United States [10] or qualified presumption of safety (QPS) in Europe [11] and with minimal risks of transferring antibiotic resistance [12]. Survivability is another key factor, as probiotics must endure both within the product and after ingestion, with strains showing resistance to acid and bile secretions and the ability to adhere to the gut epithelium displaying superior survival [13]. Probiotics should also be able to grow in bulk without genetic variation, ensuring consistent characteristics [14], and enduring the challenges of incorporation into oral delivery systems [15]. Their sensory properties should not be compromised, as the addition of probiotics should not diminish the product’s sensory quality [16]. Microbiologically, probiotics must survive in the gastrointestinal microbial ecosystem [17], and their ingestion should ideally result in no adverse side effects, such as bloating or disruptions in gut transit [18]. To enhance their survivability in the gut, probiotics must be capable of adhering to the mucosal surface [19]. Additionally, probiotics may inhibit harmful microorganisms through the production of acids, bacteriocins, or competitive exclusion [20] and have also the potential to inactivate procarcinogens, contributing to metabolic health modulation [21]. Lastly, probiotics can influence the immune system, potentially enhancing resistance to pathogens and improving outcomes related to food allergies [19].

Probiotics are normally included in food products, known as functional foods (FFs) [4]. Considering the increasing consumer demand for FF, the food industry has explored various probiotic-enriched food products, including dairy, non-dairy beverages, fermented foods, and dietary supplements. This manuscript provides valuable insights for researchers, food technologists, and policymakers involved in the development of probiotic-enriched food products. By addressing current challenges and future opportunities, it aims to contribute to the advancement of FF and public health nutrition.

## 2. Literature Search Strategy

A structured literature search was conducted to ensure a comprehensive and current review of probiotic applications in foods. Primary databases included PubMed, Scopus, Web of Science, and Google Scholar, supplemented by targeted searches using Connected Papers and ResearchRabbit. The search encompassed publications from 2000 to 2025, focusing on peer-reviewed journal articles, reviews, and relevant industry reports. Keywords included “probiotics,” “lactic acid bacteria,” “probiotic yeast,” “functional foods,” “fermented foods,” “food matrices,” “fermentation technique,” “probiotic delivery systems,” “health benefits,” “technological challenges,” “consumer acceptance,” and “regulatory considerations,” used individually and in combination with Boolean operators (AND, OR) to refine results. Additional sources were identified through cross-referencing key publications. This approach synthesizes two decades of research, providing an up-to-date, comprehensive overview of probiotic applications in diverse food systems, with emphasis on technological innovations, health benefits, and regulatory considerations.

## 3. The Health Benefits Associated with Probiotics in Food Applications

In recent decades, the incorporation of probiotic microorganisms into human diets has gained increasing attention due to their demonstrated health-promoting effects. Probiotics play a pivotal role in mitigating microbial dysbiosis, a condition implicated in the pathogenesis of various gut-associated disorders [23,24]. The prevalence of microbial imbalances has been exacerbated by increased antibiotic usage, the emergence of resistant bacterial strains, and significant dietary changes associated with modern lifestyles [25]. Their viability and metabolic activity within the gastrointestinal tract are essential for mediating a range of beneficial outcomes, including modulation of gut microbiota [23,24], enhancement of immune function [26], and prevention of gastrointestinal infections [23,24]. Table 1 presents an overview of probiotic strains, their health benefits, and associated outcomes when consumed in food.

Probiotics are commonly incorporated into both fermented and non-fermented dairy products, such as sour and fresh milk, yogurt, kefir, and cheese, which provide a favorable environment for their growth and viability [48]. These dairy products serve as primary vehicles for delivering probiotics to humans, either through independent use of probiotics as starter cultures or in combination with traditional starters. The addition of probiotics enhances the aroma, taste, texture, and health benefits of fermented dairy products [49], though challenges related to stability and effectiveness remain. In addition to dairy, other fermented beverages like Kombucha, made by fermenting tea and sugar with a symbiotic culture of bacteria and yeasts (SCOBY), have gained attention for their health benefits. Kombucha is rich in antioxidants, antimicrobial, anti-inflammatory, and anti-aging compounds, and may support immune function and help prevent diseases like diabetes, hypertension, and cardiovascular disease [50,51].

Meat fermentation with probiotic bacteria has led to the inclusion of antimicrobial peptides (e.g., bacteriocins) as an additional protective measure. Well-known fermented meat products include salami, chorizo, pepperoni, and saucisson. Fermented meat products have been proposed as suitable carriers for probiotics, which could benefit the host’s health [52]. A study showed that inulin incorporation into Frankfurt sausages affects gut microbiota and short-chain fatty acid production in rats [53]. The development of functional fermented meat products has gained interest, with innovations such as microencapsulation for probiotic protection and improvements in meat product quality [54,55]. These probiotic-enriched meat products contribute to the United Nations’ Sustainable Development Goals (SDGs), particularly promoting health and well-being, by advancing preventive medicine and potentially reducing healthcare costs [56]. However, challenges remain, including the need for careful probiotic strain selection, the stigma around the healthiness of meat products, and the limited application of probiotics in fermented sausages.

Recent research has focused on plant-based proteins, such as soybeans and peas, due to their sustainability and potential as alternatives to animal protein [57,58,59]. Soymilk, known for its health benefits, including potential prevention of chronic diseases like cancer and osteoporosis, has gained attention [60,61]. Studies have explored fermenting soymilk with LAB and bifidobacteria to enhance its health properties [62]. Fermented soy products, such as natto, miso, tofu, and tempeh, show promise in reducing gas-producing carbohydrates and increasing free isoflavones, which have various health benefits [63]. Fermented vegetables provide a suitable medium for probiotics like *Lcb. rhamnosus*, *Lcb. casei*, and *Lpb. plantarum* (formerly classified as *Lactobacillus plantarum*) [64]. Kimchi, a well-known fermented vegetable, has been found to support the viability of bifidobacteria strains [65]. Probiotics have also been integrated into cereal-based foods, which are fermented through lactic acid fermentation with yeast or molds. Cereal-based substrates can support probiotic growth and act as prebiotics due to their non-digestible components [66]. Mixed culture fermentation of oat flour with *Lpb. plantarum* has been shown to improve the viability of probiotics, making cereal-based probiotic beverages feasible [67].

Moreover, there is also an increasing interest in developing probiotic products based on fruit juice, as they provide beneficial nutrients that can serve as an ideal medium for probiotics. Fermented fruit beverages vary in characteristics depending on the fermentable sugar spectrum, probiotic strain, and nitrogen content. A study by Nguyen et al. [68] explored the fermentation of pineapple juice using *Lactobacillus* and *Bifidobacterium* strains, showing that adding prebiotic fructo-oligosaccharides increased lactic acid production and improved the stability of probiotics. *Lpb. plantarum* 299V was found to be effective for producing probiotic pineapple juice drinks. Similarly, Mantzourani et al. [69] demonstrated that fermented pomegranate juice supported the growth and viability of *Lpb. plantarum* and *Lactobacillus delbrueckii*. A more recent study by Kvakova et al. [70] showed that immobilized probiotics in banana and apple juices maintained high viability over 4 weeks, surviving in the acidic environment during lactic acid production.

Although much of the research on probiotics has focused on their application in capsules or supplements, food-based delivery systems offer an alternative approach with significant potential. Table 2 provides an overview of clinical evidence regarding the health benefits of probiotics incorporated into food products.

The clinical studies summarized in Table 2 demonstrate that probiotic efficacy is highly dependent on strain specificity, dosage, intervention duration, and the target population’s health status. While *Lcb. casei* Shirota consistently showed benefits in both gastrointestinal and stress-related outcomes, the magnitude of effects varied, with greater clinical impact observed in hospitalized or at-risk populations compared to healthy subjects [71,72]. Strains such as *Lmb. fermentum* ME-3 and *B. lactis* HN019 exhibited measurable biochemical improvements, such as reductions in LDL cholesterol and oxidative stress markers, but the small sample sizes and short intervention periods limit generalizability [75,78]. Moreover, heterogeneity in delivery formats (e.g., fermented milk vs. capsules) complicates direct comparison, as matrix effects may modulate probiotic survival and bioactivity. Collectively, these findings underscore the necessity for well-powered, long-term randomized controlled trials that standardize strain characterization, dosage, and delivery systems to validate and optimize probiotic health claims.

Although probiotics are widely recognized for their numerous health benefits, their consumption may be associated with certain mild and transient adverse effects, particularly during the initial stages of supplementation. The most reported side effects include gastrointestinal disturbances such as bloating, flatulence, diarrhea, constipation, and nausea [79,80]. These symptoms are typically self-limiting and tend to resolve within a few weeks as the host microbiome adapts to the introduced strains [79].

In addition to gastrointestinal effects, some fermented probiotic foods are rich in biogenic amines produced by probiotic LAB, such as histamine, tyramine, tryptamine, and phenylethylamine, which may trigger headaches or migraines in susceptible individuals [81]. In rare cases, probiotics may cause dermatological reactions, such as rashes or pruritus, potentially due to hypersensitivity to excipients or active components of the supplement. These symptoms usually subside upon discontinuation of the product [82].

A more serious concern, albeit infrequent, is the risk of probiotic-associated infections. Certain probiotic strains may translocate across the gastrointestinal barrier and cause opportunistic infections, particularly in immunocompromised individuals or those with severe underlying conditions. Documented clinical manifestations include bacteremia, fungemia, endocarditis, pneumonia, liver abscess, and sepsis, caused by probiotic strains such as members from the genera *Bifidobacterium*, *Lactobacillus*, *Bacillus*, and *S. boulardii* [83].

Moreover, many probiotic strains used in food or supplement formulations are genetically modified to enhance their functional properties. This raises biosafety concerns regarding potential environmental persistence, antibiotic resistance gene transfer, and unintended interactions with native microbiota. Thus, rigorous safety evaluations and regulatory oversight are required to ensure the controlled use and containment of genetically engineered probiotic strains [82,84].

Given these risks, probiotic supplementation should be approached with caution in high-risk populations, including the following: (i) individuals receiving anti-rejection medications following stem cell or solid organ transplantation; (ii) patients undergoing immunosuppressive therapy, corticosteroid treatment, or chemotherapy; (iii) individuals with autoimmune disorders or structural heart disease, particularly those with valvular abnormalities or a history of infective endocarditis; (iv) patients with acute abdominal conditions, active intestinal diseases (e.g., colitis), neutropenia, or those at risk of intestinal perforation [82,85]. In such cases, the potential benefits of probiotic administration must be carefully weighed against the possible risks, and clinical use should be guided by evidence-based protocols and patient-specific considerations.

## 4. Different Fermentation Techniques and Food Matrices Used for Probiotic Incorporation

Functional foods enriched with probiotics are increasingly recognized for their role in promoting gastrointestinal health, enhancing immune function, and potentially preventing chronic diseases. The successful delivery of probiotics through food systems relies on suitable fermentation processes and compatible food matrices that maintain microbial viability and bioactivity during production, storage, and gastrointestinal transit.

### 4.1. Fermentation Techniques for Probiotic Incorporation

Traditional spontaneous fermentation represents a traditional process by which probiotic microorganisms are naturally incorporated into food matrices without the intentional addition of defined starter cultures. In some cases, enzymatic rennet may be employed to aid coagulation, but microbial succession remains predominantly driven by native microbiota [4,86,87]. In such systems, the metabolic activity of indigenous microbiota present in raw materials or the processing environment drives fermentation, with LAB such as species from genera *Lactobacillus*, *Leuconostoc*, and *Pediococcus*, alongside yeasts including species from genera *Saccharomyces* and *Kluyveromyces*, playing a dominant role [88,89]. These indigenous microbes often exhibit probiotic potential, including the ability to survive gastrointestinal transit, modulate the host immune response, and compete against pathogens [4]. Spontaneous fermentation not only enhances the nutritional and sensory attributes of the final product but also enriches it with a diverse microbiota that may confer health benefits. This process underpins the production of a wide range of traditional fermented foods, including cheeses [86,90,91], *kimchi* [92], *kefir* [93], *kombucha* [94], and African foods such as *ogi* and *masa* [95]. However, the uncontrolled nature of spontaneous fermentation can lead to variability in probiotic content and viability, highlighting the need for careful monitoring and characterization of microbial communities to ensure safety and functional efficacy in probiotic delivery.

Controlled batch fermentation is a standardized method for the targeted incorporation and production of probiotic microorganisms under reproducible and tightly regulated conditions. Unlike spontaneous fermentation, which depends on naturally occurring microbiota, this approach employs intentionally selected and well-characterized probiotic strains, such as species from genera *Lactobacillus*, *Bifidobacterium*, or *S. boulardii*, as starter cultures. The fermentation process is carried out in closed bioreactors or fermenters, where critical parameters including pH, temperature, oxygen levels, and nutrient composition are continuously monitored and optimized to maximize microbial growth, viability, and metabolic activity [72]. The use of defined starter cultures under controlled conditions ensures product consistency, safety, and a predictable probiotic load, which are essential for maintaining functional efficacy and meeting regulatory requirements. Controlled fermentation also enables reproducible delivery of health benefits associated with specific strains, addressing the variability and unpredictability of traditional spontaneous processes. Well-established examples include yogurt production using *S. thermophilus* and *Lb. delbrueckii* subsp. *bulgaricus* [96] and probiotic-enriched fermented milk employing *Lcb. casei* Shirota [97]. Due to its ability to minimize variability and ensure product safety and quality, controlled batch fermentation represents the predominant method in industrial probiotic food manufacturing and provides a reliable platform for the large-scale development of functional foods. *Immobilized cell fermentation* is an advanced biotechnological approach for the incorporation and production of probiotic microorganisms, wherein viable cells are physically confined or attached to a solid support matrix, without losing metabolic activity. Common immobilization carriers include natural polymers (e.g., alginate, carrageenan, chitosan), synthetic polymers, porous beads, or food-grade materials. This technique offers several advantages over traditional free-cell fermentation, including enhanced cell stability, higher cell density, extended viability during storage, and improved resistance to environmental stresses such as low pH or bile salts. Immobilized probiotic cells can be used in both batch and continuous fermentation systems, facilitating the sustained release of live microorganisms and bioactive metabolites. This method is particularly beneficial for FF production, allowing for the development of probiotic-enriched products with improved shelf life and targeted delivery. Moreover, immobilized cell systems support repeated or continuous use of probiotic cultures, improving process efficiency and reducing production costs. Due to these benefits, immobilized cell fermentation is increasingly applied in dairy, non-dairy, and pharmaceutical probiotic formulations [98].

Co-fermentation techniques involve the simultaneous cultivation of two or more microbial species, typically probiotic strains alongside other functional or fermentative microbes, to enhance the nutritional, sensory, and functional properties of fermented products. In the context of probiotic incorporation, co-fermentation allows beneficial microorganisms (probiotics such as *Lactobacillus*, *Bifidobacterium*, or *S. boulardii*) to be grown in synergy with other lactic acid bacteria, yeasts, or even prebiotic-degrading microorganisms. These mutualistic relationships can improve probiotic viability, metabolic activity, and gastrointestinal stability, while also contributing to product consistency and functionality [99]. For instance, the co-fermentation of probiotic *Lactobacillus* spp. with *S. thermophilus* and *Lb. delbrueckii* subsp. *bulgaricus* in yogurt not only accelerates acidification kinetics and enhances texture but also supports probiotic survival during fermentation and storage [5]. Similarly, kefir grains provide a naturally occurring co-fermentation system, comprising lactic acid bacteria, acetic acid bacteria, and yeasts, which together drive the production of lactic acid, ethanol, and diverse bioactive metabolites [100]. Co-fermentation of soy milk with *Lpb. plantarum*, *Lb. acidophilus*, and *Lb. bulgaricus* has also been shown to improve both probiotic viability and nutritional quality [101]. However, in co-fermentation systems, the inoculation strategy is a critical determinant of process efficiency and probiotic survival. The ratio and functional compatibility between probiotic strains and traditional starter cultures must be carefully optimized, as imbalances can lead to antagonistic interactions, competition for nutrients, or inhibitory metabolite production, ultimately reducing probiotic viability and product quality [102]. Beyond microbial survival, co-fermentation enhances the production of bioactive compounds, including short-chain fatty acids, peptides, vitamins, and exopolysaccharides, which enrich the health-promoting potential of the final product [99]. This approach is particularly valuable in the development of multifunctional foods, where the combined action of different microorganisms contributes not only to improved probiotic delivery but also to flavor development and overall product quality. *Synbiotic fermentation* involves the simultaneous use of probiotics (beneficial live microorganisms) and prebiotics (non-digestible food ingredients that selectively stimulate probiotic growth) during the fermentation process. This strategy enhances the effectiveness of probiotic incorporation by supporting the viability, metabolic activity, and colonization potential of probiotic strains in both the food matrix and the gastrointestinal tract. In synbiotic systems, prebiotics such as inulin, fructooligosaccharides (FOSs), and galactooligosaccharides (GOSs) act as fermentable substrates for probiotics. The resulting lactic acid fermentation not only preserves the food but also creates a favorable environment that protects probiotics against adverse conditions such as oxygen exposure, low pH, and bile salts. González-Herrera et al. [103] summarized the FF where probiotics are used with prebiotics. For example, inulin, commonly used for its prebiotic effects, also improves texture, taste, and moisture retention of many foods [104]. Studies have demonstrated comparable or superior sensory performance in probiotic products such as yogurt with *Lmb. reuteri* RC-14, *Lcb. rhamnosus* GR-1, and insulin [105], chocolate mousse with *Lacticaseibacillus paracasei* (formerly classified as *Lactobacillus paracasei*) [106], and milk fermented with *B. animalis* and *Lb. acidophilus* La-5 and supplemented with inulin [107]. However, sugar levels in products like fruit yogurt must be carefully controlled, as levels above 10% can negatively affect consumer acceptance [108,109].

The prebiotic source appears to strongly influence both sensory and functional outcomes [110]. For instance, Mobasserfar et al. [111] reported that high-methoxyl grape pomace pectin (GPP) supplementation in low-fat set-type yogurt enhanced sensory attributes and significantly improved the survival of *Lb. acidophilus* LA-5 compared to *B. bifidum* BB-12, with probiotic counts remaining higher than in pectin-free controls during storage. In contrast, Muniz Pereira et al. [112] found that FOS addition to skyr yogurt did not significantly affect physicochemical or sensory characteristics, though probiotic viability was not assessed. Similarly, Allgeyer et al. [113] observed that inulin, soluble corn fiber, and polydextrose modified the sensory properties of yogurt drinks, with additional changes upon probiotic inclusion (*B. animalis* subsp. *lactis* Bb-12 and *Lb. acidophilus* LA-5); however, probiotic counts decreased by 2–3 logs after 30 days of refrigerated storage, regardless of prebiotic type. Beyond inulin-based ingredients, some fruit-derived fibers show promise for probiotic protection. Dimitrellou et al. [114] demonstrated that incorporating apple fibers, an apple processing by-product with prebiotic and technological benefits, into yogurt significantly improved the survival of *Lcb. casei* ATCC 393 during production, storage, and simulated gastrointestinal digestion compared to the yogurt matrix alone. Collectively, these findings indicate that the impact of prebiotics on probiotic viability is not universal but is influenced by factors such as molecular structure, water-holding capacity, ability to interact with bacterial cell surfaces, and potential effects on microenvironmental pH. Therefore, prebiotic selection in synbiotic dairy formulation should be guided by both sensory performance and strain-specific survival data, rather than sensory considerations alone. Table 3 summarizes the pros and cons of different fermentation techniques used for probiotic incorporation in food.

### 4.2. Food Matrices for Probiotic Delivery

The food matrix plays a critical role in the successful delivery, viability, and functionality of probiotics in FF products. A well-designed matrix not only ensures the survival of probiotics during processing, storage, and gastrointestinal transit, but also influences the sensory properties, consumer acceptance, and efficacy of the final product.

Dairy-based products, including fermented milk, yogurt, cheese, infant formula, and ice creams, remain the most established carriers for probiotics due to their optimal physicochemical properties and nutrient-rich composition. These matrices buffer gastric stressors and supply proteins, peptides, and lipids that support probiotic viability. Semi-solid textures further enhance gastrointestinal retention, while semi-hard and hard cheeses offer superior microbial stability owing to low moisture content and extended shelf life [103]. Dairy matrices like cheese and fermented milk offer protective effects for probiotics due to their pH buffering capacity and fat content, enhancing microbial survival through the gastrointestinal tract [120]. Functional yogurts enriched with vitamins, minerals, sterols, prebiotics, and probiotics have demonstrated sustained market success [24].

Recent studies highlight several health benefits of probiotic dairy products, including improved mineral absorption, anti-*H. pylori* activity, and prevention of gastrointestinal disorders [49]. For instance, *Lpb. plantarum* has shown hypocholesterolemic effects and resistance to gastrointestinal stress [121], while *Lcb. rhamnosus* has demonstrated antimicrobial activity against *Listeria monocytogenes* without compromising cheese quality [122]. Moreover, strains like *Leuconostoc mesenteroides* CM9 and *Lb. acidophilus* have exhibited strong immunomodulatory and antimicrobial properties [123,124]. *S. boulardii* CNCM I-745 has been effectively incorporated into synbiotic yogurt formulations, particularly in combination with prebiotics like inulin, demonstrating favorable quality and probiotic viability [125]. These findings underscore the therapeutic potential and technological advancements in probiotic dairy applications.

Other animal-derived matrices, such as meat and eggs, are emerging as alternative probiotic vehicles. Fermented sausages, cooked meats, and ready-to-eat products provide high protein and fat content conducive to microbial survival, yet processing variables (e.g., temperature, salt, curing agents) must be carefully controlled. Meat products constitute a fundamental nutritional source, with raw, ripened, and cured meats traditionally fermented to enhance shelf life and flavor. Fermentation generates metabolites, including lactic acid, pyruvic acid, alcohols, aldehydes, ketones, and carboxylic acids, which influence meat quality and preservation. Proteolysis during fermentation degrades muscle proteins into peptides and free amino acids, contributing to improved aroma, color, and taste [126]. Certain probiotics also produce bioactive compounds like conjugated linoleic acid (CLA), which modulates immune and inflammatory responses and confers cardiovascular benefits. Fermented meats, such as sausages, are often uncooked or ready-to-eat, making them effective probiotic delivery vehicles by embedding microorganisms within the protein and fat matrix. Studies report significant increases in probiotic counts during fermentation; for instance, *Lactobacillus* inoculated at 10^5^ CFU/g can reach 10^9^ CFU/g post-fermentation. In mutton sausages supplemented with *Lb. acidophilus* CCDM 476 and *Bifidobacterium animalis* 241a, *Lb. acidophilus* exhibited superior viability over 60 days, alongside improved texture and aroma [127,128]. Despite these benefits, probiotic incorporation in meat faces challenges related to low water activity, limited sugar availability, and complex indigenous microbiota. Therefore, probiotic strains must be specifically selected for their resilience in meat environments and fermented product conditions to maintain efficacy [129].

Currently, dairy-based products remain the predominant probiotic carriers, owing to their favorable nutrient composition and buffering capacity. However, rising demand for non-dairy alternatives stems from lactose intolerance, dairy allergies, and vegan or vegetarian dietary preferences. Non-dairy probiotic foods offer advantages such as reduced allergenicity and lower cholesterol content, which may benefit cardiovascular health and adiposity reduction [126].

Plant-based matrices are increasingly favored in response to demand for vegan, lactose-free, and allergen-free alternatives. Diverse substrates, including cereals, legumes, fruits, vegetables, and plant-based beverages (e.g., soymilk, coconut milk, rice milk, oat milk, almond milk, walnut milk, Quinoa milk, hazelnut milk, cashew milk, hemp milk, maize milk), offer unique nutritional benefits [129]. However, maintaining probiotic viability in these matrices may require fortification with prebiotics or encapsulation techniques to overcome fermentation and storage challenges [130]. Plant-based matrices, including fruit and vegetable juices, purees, pulps, beverages, and dried products, offer rich sources of carbohydrates, fibers, vitamins, polyphenols, and minerals that promote probiotic viability and health benefits [129]. Aqueous extracts of fruits such as kiwifruit and avocado have demonstrated low cytotoxicity and enhanced anti-inflammatory effects, while non-aqueous forms may show higher cytotoxicity, but also stronger anti-inflammatory potential [131]. Combining probiotics with micronutrients (calcium, vitamins, carotenoids) may further enhance functional properties, presenting promising avenues for probiotic juice development [132,133,134]. However, the typically low pH of fruit juices challenges probiotic survival. Acid-tolerant strains like *Lpb. plantarum*, *Lb. acidophilus*, and *Lcb. casei* demonstrate better viability, whereas *Bifidobacterium* strains are more sensitive and often fail to survive at pH below 4.6 [129,131]. The viability of probiotics is shorter in non-dairy foods when compared to dietary supplements due to the harsh environments faced by probiotics in beverages. Processors must consider many factors in the production of probiotic juices, such as pH, temperature, anthocyanins, and most importantly, a vegetative form of probiotics [135]. Maintaining cell viability and shelf stability remains essential for the effectiveness of probiotic-enriched juices.

Cereal grains such as wheat, maize, oats, and barley are rich in dietary fibers and bioactive compounds that nourish the gut microbiota by providing fermentable carbohydrates serving as prebiotics. Additionally, cereals act as encapsulation matrices, enhancing probiotic survival. Whole grains contain phytochemicals, including antioxidants, phytic acid, sterols, phenolics, and phytoestrogens. Fermentation improves mineral bioavailability by enzymatic phytate degradation and creates an optimal pH for lactic acid bacteria growth. Fermented cereal products thus provide combined prebiotic and probiotic benefits similar to fermented dairy products. Notably, cereals like oats and barley, with high β-glucan content, exhibit hypocholesterolemic effects, supporting their use as probiotic carriers in functional foods [129].

Bakery products, rich in essential nutrients such as carbohydrates, proteins, and fibers, are being explored as potential carriers for probiotics [136]. However, the high temperatures involved in baking pose a significant challenge to probiotic viability. Recent advances focus on incorporating thermo-resistant probiotic strains and protective delivery systems, such as microencapsulation and sourdough fermentation, to enhance survival during baking and gastrointestinal transit [137]. Encapsulation of *Lcb. rhamnosus* in sodium alginate and *Lpb. plantarum* using matrices like gum arabic and skim milk has shown promising results in maintaining cell viability post-baking [138,139]. Additionally, lower pH during storage was found to support increased probiotic survival. These strategies indicate that bakery products can serve as viable functional foods with improved probiotic delivery.

Confectionery and snack foods (e.g., chocolate, gummies, bars) represent innovative delivery systems for probiotics, appealing to consumer preferences for convenience and taste. For example, high-fat content in products like chocolate can provide protective effects against oxidative and acid-induced stress, though specialized processing is necessary to preserve microbial integrity throughout production and shelf life [140].

A recent study by Mohan et al. [141] explored the effect of Manuka honey on probiotic growth, survival, and activity in synbiotic food matrices, finding that incorporating 5% Manuka honey could support desired probiotic counts (over 8 log cfu/mL for *Limosilactobacillus reuteri* DPC16, formerly classified as *Lactobacillus reuteri*) in yogurt. These authors suggested that the combination of *Lmb. reuteri* DPC16 and AMF15+ Manuka honey could be developed into a synbiotic functional food. Similarly, Jan Mei et al. [142] showed that adding 5% Tualang and Tapah honey enhanced the growth of *Bifidobacterium longum* BB 536. ŽugićPetrović et al. [143] suggest that incorporating probiotic starter cultures (*Latilactobacillus curvatus* sk1-8 (formerly classified as *Lactobacillus curvatus*), *Latilactobacillus sakei* IIb1 (formerly classified as *Lactobacillus sakei*), and *Staphylococcus xylosus* sk-Ios4 and organic sunflower honey (0.2%) enhances both the functional and sensory properties of Sokobanja sausage, offering a promising approach for improving quality and safety. This probiotic growth enhancement is likely due to the unique oligosaccharide components and antibacterial properties of honey.

Therefore, the selection of an appropriate food matrix is crucial for the development of efficacious probiotic FFs. Each matrix presents unique advantages and limitations regarding probiotic viability, technological feasibility, and consumer appeal. Table 4 summarizes the key features, advantages, and challenges of different matrices used for probiotic delivery.

## 5. Technological Challenges in Probiotic Stability and Viability During Processing and Storage

Probiotics play a pivotal role in the development of FFs due to their documented health-promoting properties. However, the successful incorporation of viable probiotic cells into food matrices remains technologically challenging, particularly during processing and storage [119]. The survival and functionality of probiotics depend on a complex interplay of intrinsic food properties, processing parameters, and microbiological factors. Optimizing these variables is essential to maintain microbial viability and deliver efficacious doses in commercial products.

### 5.1. Processing Conditions Influencing Probiotic Viability

The viability and functionality of probiotics in food matrices are strongly influenced by both intrinsic and extrinsic factors, including temperature, pH, titratable acidity, oxygen availability, water activity (A_w_), and the presence of salts, sugars, and antimicrobial compounds (e.g., hydrogen peroxide, bacteriocins, and synthetic preservatives). Oxygen exposure and antimicrobial agents are known to reduce microbial survival, whereas moderate concentrations of salts and sugars may exert protective effects by stabilizing microbial cells. For instance, studies have demonstrated that oxygen availability significantly impacts the growth, fermentation activity, and stability of *S. boulardii* and *Kluyveromyces lactis* in probiotic formulations, emphasizing the necessity of optimized oxygen conditions during production and storage [166,167]. Processing-related factors, such as inoculation temperature, incubation duration, thermal treatments, cooling rates, storage temperature, packaging permeability, and production scale, further modulate probiotic viability [22,168,169]. Fermentation at 37 °C supports probiotic proliferation in plant-based substrates, whereas high-temperature treatments such as pasteurization markedly reduce viability; therefore, the use of thermotolerant strains (e.g., *Lb. acidophilus* NCFM, *S. boulardii*) and post-processing inoculation are effective strategies to maintain functionality [170,171,172]. Optimal growth for most LAB occurs between 30 and 45 °C, although strain-specific differences exist: mesophilic LAB thrive at 20–25 °C in meat fermentations, followed by maturation at 12–15 °C, while thermophilic species such as *Lb. acidophilus* and *S. thermophilus* tolerate up to 45 °C but exhibit metabolic impairment beyond this range [119,173,174,175]. Excessive salt concentrations (>3% *w*/*w*) impose osmotic stress, yet moderate levels enhance flavor and stability; thus, dairy systems typically apply 1.5–2% *w*/*w* salt, while dry-cured meats use higher concentrations (3.5–5%), necessitating the employment of salt-tolerant strains such as *Lpb. plantarum* and *Pediococcus pentosaceus* [176,177,178]. Additionally, microbiological parameters such as strain selection, resistance to oxidative and acidic stress, inoculation levels, and compatibility with native or co-cultured microorganisms are critical for maintaining probiotic populations throughout product shelf life [22,179].

### 5.2. Matrix-Specific Considerations

The food matrix plays a pivotal role in determining probiotic survival and functionality. Dairy-based systems, such as yogurt and fermented milk, provide a nutrient-rich and buffered aqueous environment that supports the growth of common probiotics, including *Lb. delbrueckii* ssp. *bulgaricus* and *S. thermophilus*, although pH, acidity, and oxygen levels must be carefully controlled [24,180]. In contrast, fermented meat products present a high-protein, high-fat matrix with low water activity and acidic pH, which may protect probiotics but also impose challenges due to the presence of curing agents and competitive native microbiota [143,175]. Plant-based substrates exhibit greater variability, with pH, polyphenols, and organic acids exerting strain-dependent inhibitory or stimulatory effects, while carbohydrate composition, particularly the balance of simple sugars and complex fibers, critically influences fermentability and probiotic persistence [159]. The application of curing agents is matrix-specific: dairy systems rely mainly on antimicrobial metabolites naturally produced by starter cultures, plant-based substrates benefit from natural preservatives such as garlic, ginger, or nitrate-rich vegetables [4,181], and meat fermentations frequently employ sodium nitrite, ascorbic acid, and sucrose to enhance microbial safety, stabilize sensory properties, and promote probiotic dominance [182,183].

### 5.3. Effects of Processing and Storage

Processing and storage steps exert a dual influence on probiotic viability and functionality. When carefully optimized, these processes can promote microbial growth, extend survival during shelf life, and preserve functional activity. Conversely, inappropriate conditions may compromise cell integrity and reduce probiotic efficacy, underscoring the need for system-specific adaptation. In dairy matrices, critical parameters include fermentation temperature and duration, pasteurization, cooling regimes, and packaging under conditions with low oxygen permeability. Meat-based systems present additional challenges such as heat treatment during cooking, mechanical stress from grinding or mixing, and exposure to curing salts or modified atmospheres. Plant-derived matrices often involve pasteurization or sterilization, mechanical homogenization, and the addition of preservatives, all of which necessitate strain-specific optimization. Confectionery products, which typically undergo high-temperature treatments such as tempering, baking, or roasting, require advanced protective strategies, including microencapsulation or lipid-based coatings [180,184].

Storage conditions are equally critical for maintaining probiotic viability. Cold chain integrity, oxygen exclusion, pH stability, and moisture control are central determinants of survival. Oxygen exposure, for instance, can generate reactive oxygen species that damage cellular membranes, whereas repeated freeze–thaw cycles impose osmotic stress that disrupts cell integrity. Packaging materials also influence stability; polyethylene, for example, permits oxygen diffusion and may compromise microbial survival unless supplemented with oxygen scavengers or antioxidants such as catechins [180,184].

Overall, processing and storage conditions are not inherently detrimental or beneficial. Their effects depend on the complex interplay between the technological parameters, the food matrix, and the specific probiotic strain. Optimized procedures can preserve, and in some cases enhance, probiotic viability and functionality, whereas unoptimized processes can significantly diminish their beneficial properties.

### 5.4. Probiotic Survival and Functional Efficacy

Probiotic efficacy varies considerably across different food matrices due to differences in composition, processing, and storage conditions. In dairy products such as yogurt, cheese and fermented milk, studies report maintenance of 10^8^–10^9^ CFU/mL of *Lactobacillus* and *Bifidobacterium* strains over typical shelf-life periods [86,87,90,185], whereas in plant-based beverages, survival often declines to 10^6^–10^7^ CFU/mL under similar storage conditions, likely due to lower buffering capacity and the presence of inhibitory polyphenols [186]. In fermented meat products, probiotic counts of *Lpb. plantarum* and *P. pentosaceus* have been reported to remain between 10^7^ and 10^8^ CFU/g, although exposure to curing salts and low water activity can reduce viability by 1–2 log units [175,187]. Co-fermented and symbiotic systems often demonstrate improved probiotic stability, with effect sizes ranging from 0.5 to 1 log higher viable counts compared to mono-culture fermentations, likely due to synergistic microbial interactions and prebiotic supplementation [143]. Overall, these findings highlight that probiotic survival and functional efficacy are highly matrix-dependent, and strategies such as strain selection, encapsulation, and optimization of processing and storage conditions are critical to maximizing health benefits across different food products.

### 5.5. Encapsulation and Smart Delivery Systems for Enhancing Probiotic Viability in Functional Foods

The successful development of functional foods (FFs) containing probiotics requires careful selection of food matrices and fermentation techniques, alongside the implementation of advanced strategies that ensure probiotic viability during processing, storage, and gastrointestinal transit [119]. Among these, encapsulation and smart delivery systems have emerged as critical technologies, as they provide both physical and functional protection for probiotic microorganisms and bioactive molecules. By creating a protective microenvironment, these systems mitigate the detrimental effects of environmental stresses such as oxygen exposure, low pH, temperature fluctuations, and mechanical forces, thereby extending product shelf-life and enhancing the likelihood of delivering sufficient viable counts to the colon, a prerequisite for health benefits [188,189]. Encapsulation and immobilization of probiotics have been extensively applied in both dairy and non-dairy systems to improve microbial viability, stability, and functional performance. Their selection is largely determined by the physicochemical characteristics of the food matrix, the target consumer group, and the desired shelf-life. Furthermore, these technologies have been shown to improve not only probiotic survival but also the sensory properties and consumer acceptance of probiotic-enriched products [190,191,192]. Consequently, encapsulation and immobilization represent key enablers in the design of next-generation functional foods, ensuring both technological feasibility and consumer-driven efficacy [191].

#### 5.5.1. Encapsulation Technologies and Materials

Encapsulation involves entrapping probiotic cells within a protective matrix, serving as a barrier against environmental stressors such as heat, oxygen, moisture, pH fluctuations, and enzymatic degradation. A range of biopolymers is employed as encapsulating agents, including alginate, chitosan, gelatin, whey protein, carrageenan, and various polysaccharides and lipids. The choice of encapsulation material and technique is largely dictated by the physicochemical properties of the probiotic strain, the food matrix, and the intended release profile [193].

The most commonly used conventional encapsulation techniques are as follows: (i) Spray drying is valued for its low cost, high productivity, and scalability. Despite these advantages, the high temperatures used can compromise cell viability, and the resulting particles often have porous surfaces that may affect stability and release characteristics [193,194,195]; (ii) Spray cooling generates dense, spherical particles and is similarly cost-effective, but it is less suitable for thermosensitive compounds due to potential bioactive losses [196,197]; (iii) Freeze drying (lyophilization) is ideal for heat-sensitive strains, offering mild dehydration and high retention of functional properties. However, it is expensive, difficult to scale, and may lead to cell damage and reduced viability [196]; (iv) Fluidized bed coating enables large-scale production and is suitable for thermally sensitive materials. The process, however, is technically complex and costly [193,194]; (v) Extrusion is a mild and cost-effective method but produces large particles with lower microbial viability and has limited scalability. It also necessitates the use of hydrocolloid solutions for encapsulation [194]; (vi) Ionic gelation is a straightforward and solvent-free technique that operates under gentle conditions but often results in non-uniform particle sizes [197]; (vii) Emulsification provides high encapsulation efficiency and is flexible but is not optimal for large-scale production and typically yields non-uniform particles [194].

#### 5.5.2. Innovative and Smart Encapsulation Strategies

Recent innovations focus on smart delivery systems that improve targeted release and functional performance. Encapsulation using yeasts is a novel technique offering high mechanical strength, encapsulation efficiency, and scalability. This cost-effective method eliminates the need for additional materials but is currently limited to lipophilic substances [198]. Inclusion complexation, especially with β-cyclodextrin, has shown promise for volatile compound stabilization and controlled release. However, high cyclodextrin concentrations may raise health concerns, requiring careful formulation [197,198]. Smart delivery systems utilize stimuli-responsive carriers that release probiotics in response to environmental cues such as pH changes in the gastrointestinal tract. pH-sensitive hydrogels, typically composed of protein–polysaccharide composites, are biodegradable and biocompatible. For example, Liu et al. [199] demonstrated that soy protein isolate–citrus pectin hydrogels, crosslinked with transglutaminase and treated with ultrasound, significantly enhanced the survival of *Lpb. plantarum* under simulated gastrointestinal conditions. Liposomes allow the encapsulation of both hydrophilic and hydrophobic probiotics, producing uniform particles without organic solvents. Despite these benefits, they are thermodynamically unstable, posing risks of premature release [193,200]. Nanoencapsulation represents a cutting-edge approach, offering superior stability and targeted delivery. A variety of techniques are employed, including antisolvent precipitation, electrospinning, electrospraying, high-pressure microfluidization, nanoemulsification, and ultrasonication-assisted emulsification. Although nanoencapsulation generally entails higher production costs than traditional techniques such as spray drying, spray cooling, or freeze-drying, primarily due to the need for specialized equipment, precise process control, and high-quality raw materials, it provides distinct functional advantages that may justify its use in specific applications. Despite these economic considerations, nanoencapsulation offers distinct functional advantages that can justify its application in targeted scenarios. Notably, nanoencapsulated probiotics demonstrate enhanced stability under adverse processing and gastrointestinal conditions, improved targeted delivery, and increased bioavailability compared to macroscale encapsulated systems [193]. Furthermore, nanoencapsulation enables controlled release of probiotics and bioactive compounds, potentially enhancing efficacy at lower dosages. While conventional encapsulation remains more economically favorable for large-scale applications, nanoencapsulation offers strategic benefits for high-value, precision-targeted functional foods and nutraceutical products [201,202]. Finally, the selection of an encapsulation technique for probiotics must be guided by the specific requirements of the food application, target release site, and stability profile, balancing technological feasibility with probiotic functionality and consumer safety. These methods enhance the stability and bioavailability of probiotics and enable their co-delivery with other bioactive substances, including prebiotics (e.g., inulin, polydextrose, FOS, GOS), omega-3 fatty acids, or multiple probiotics, thereby improving both microbial viability and functional performance [193].

#### 5.5.3. Applications of Immobilization and Encapsulation in Food Systems

Probiotic cell immobilization and encapsulation techniques have been extensively applied in various food systems to enhance the viability, stability, and functionality of probiotic microorganisms. Encapsulation has emerged as a promising strategy to enhance probiotic stability and functionality in food systems. Hydrocolloids such as sodium alginate, carrageenan, and whey protein isolates are commonly applied to protect cells from adverse environmental conditions, including heat, oxygen exposure, and acidic pH, thereby improving survival during industrial processing and storage [203,204]. More advanced systems employ lipid-based carriers and protein–polysaccharide complexes, which form multilayered protective barriers that further enhance resistance to physicochemical stresses [201,205,206]. In addition, micro- and nanoencapsulation technologies enable targeted and controlled release in the gastrointestinal tract, typically triggered by pH fluctuations or enzymatic activity [193].

In dairy applications, immobilization of *Lcb. casei* ATCC 393 on casein and apple pieces has demonstrated high survival rates during refrigerated storage [207], while alginate encapsulation of *Lcb. casei* improved viability and sensory properties in fermented dairy products [208]. Sodium alginate-immobilized *Lcb. casei* has also been used in synbiotic milk chocolate [209], and probiotic yogurt has been enriched by incorporating *Lcb. casei* and *Lb. delbrueckii* subsp. *bulgaricus* applied to fruits and oat pieces [210]. Encapsulation of *Lb. acidophilus* in calcium alginate has been shown to enhance its survival in yogurt [211], and whey protein–alginate combinations have been employed to protect *S. thermophilus*, *Lb. delbrueckii* subsp. *bulgaricus*, *Lb. acidophilus*, and *B. bifidum* during gastrointestinal transit [212]. Additionally, whey protein has served as a protective matrix for *Lcb. rhamnosus* in products including biscuits and vegetable juice blends [213]. Additional studies highlight more specialized encapsulation strategies. For instance, *Lcb. casei* ATCC 393 in fermented milk was stabilized with Chios mastic gum through freeze-drying, resulting in improved viability and functional properties [119]. Similarly, *Lb. acidophilus* has been delivered in yogurt using a pectin–whey protein matrix through ionic gelation and complexation [214] and in yogurt–ice cream products via sodium alginate extrusion [215].

In non-dairy systems, encapsulation technologies have enabled the fortification of beverages, bakery products, meat products, and plant-based alternatives with viable probiotics. For example, *Lpb. plantarum* LBRZ12 has been incorporated into mayonnaise with dill and basil essential oils via alginate immobilization [216], while chitosan-coated alginate beads have been developed to protect *Lcb. casei*, *Lb. acidophilus*, and *Lpb. plantarum* against gastrointestinal conditions [217]. Fibrous carriers have been used to deliver *Lcb. rhamnosus* and *Lb. acidophilus* in apple juice and chocolate-coated cereals [218], and calcium alginate–based gels have been applied to fortify bread with *Lcb. paracasei* [219]. The exploitation of fruit waste (e.g., rinds of durian, mangosteen, and jackfruit) as natural immobilization matrices for *Lb. acidophilus* and *Lb. bulgaricus* in soy milk [220]. Other examples include *Lmb. reuteri* in fermented sausages [221], *Lb. acidophilus* in tomato juice [222], and *Lpb. plantarum* in mango or pomegranate juice using alginate or chitosan-based coatings [223,224].

Comparative analysis reveals that dairy matrices, with their buffering capacity and nutrient-rich composition, often allow for simpler encapsulation systems such as alginate, whey protein, or pectin–protein complexes, relying on extrusion or freeze-drying for stability. In contrast, non-dairy matrices, often more acidic, oxidative, or thermally processed, tend to require multi-layered or composite encapsulation systems (e.g., alginate–chitosan, alginate–soy protein isolate, or poly-γ-glutamic acid) to maintain probiotic viability. This distinction underscores how encapsulation choice is closely tailored not only to the food type but also to processing conditions, storage stability requirements, and the targeted consumer segment. These applications highlight the versatility and efficacy of immobilization strategies for delivering viable probiotics across a broad range of food matrices.

## 6. The Impact of Probiotics on Consumer Acceptance of Food Products

The incorporation of probiotic microorganisms into food products is driven by increasing consumer awareness of gut health and demand for functional foods with added health benefits. However, the success of probiotic-enriched products in the market is not solely dependent on their health-promoting attributes, but also on their sensory appeal and consumer acceptance. Probiotic microorganisms have been indicated to be present in the food at minimum concentrations of log 6–7 CFU/g, with the daily therapeutic dose being about log 8–9 CFU/g [225], which is a high number that allows them to survive the gastrointestinal conditions. The addition of probiotics may lead to alterations in the physicochemical and organoleptic characteristics of food, including changes in taste, texture, aroma, color, and overall palatability, which can significantly influence consumer perception and purchase intent.

The interaction between yeast strains, particularly *Saccharomyces cerevisiae* var. *boulardii*, and LAB plays a significant role in shaping the sensory characteristics of fermented foods. *S. cerevisiae* secretes amino acids and carbon dioxide, which not only support LAB growth but also influence the aroma and flavor profile of the final product. The release of carbon dioxide by yeast creates micro-anaerobic zones favorable for LAB metabolism, indirectly enhancing lactic acid production, which contributes to the sourness and mouthfeel of fermented dairy products [226]. Additionally, the amino acids released by yeast, such as threonine, glutamine, and serine, are precursors to aroma-active compounds formed through microbial catabolism during fermentation. These compounds contribute to the complexity and acceptability of flavor in symbiotic and fermented food matrices [227]. In products like kefir, lactic acid not only imparts acidity but can also be metabolized by LAB under aerobic conditions to produce biopolymers like kefiran, which affects texture and viscosity, key sensory attributes valued by consumers [228]. Exopolysaccharides (EPSs) from food-grade LAB serve as non-toxic, biodegradable agents with functional roles as natural thickeners, stabilizers, and emulsifiers. EPS can be applied to starch-based foods to improve texture and prevent syneresis. For instance, adding 1% EPS to wheat starch reduced syneresis by 50% and increased viscosity by 28% [229].

Probiotic microorganisms not only provide health benefits but also influence the physicochemical and sensory characteristics of food products, particularly texture. Several studies described by Guimarães et al. [230] have examined how different probiotics impact texture in a variety of food matrices. Briefly, in ice cream, the incorporation of *Lcb. casei* 01 resulted in products with reduced fat destabilization and a notable increase in apparent viscosity, although the melting time was shortened. Importantly, *Lcb. casei* 01 remained viable at a therapeutic level (>6 log CFU/mL) over five months of storage, supporting both functional and structural roles in frozen dairy products [231]. In the case of fresh cheeses, such as panela cheese, the use of *Lcb. rhamnosus* GG and *Bifidobacterium breve* improved textural attributes. Products containing *Lcb. rhamnosus* GG were rated higher by consumers for compactness, hardness, moisture, and softness, indicating that this strain contributes positively to the overall mouthfeel and acceptance of the cheese [232]. For cream cheese, the addition of encapsulated and non-encapsulated *Lcb. rhamnosus* demonstrated different textural outcomes. Encapsulated probiotics significantly increased firmness compared to non-encapsulated ones. Initially, non-encapsulated probiotics led to a softer texture, but this effect diminished after 21 days of storage, while firmness increased in samples with encapsulated probiotics. These changes were attributed to acid development and proteolysis during storage [233]. In fruit-based matrices like orange juice, the use of sodium alginate-encapsulated *Lcb. casei* 01 altered the rheological profile, resulting in products with a higher consistency index and lower flow behavior index. However, these structural modifications led to lower consumer acceptance, emphasizing the importance of balancing functional and sensory properties [234]. In vegetable-based products such as carrot purée, the fermentation with exopolysaccharide-producing lactic acid bacteria (e.g., *Leuconostoc lactis*, *Weissella confusa*) led to significant changes in texture. These strains produced thicker and more palatable products. Although not yet officially recognized as probiotics, these bacteria show promising potential for enhancing texture in plant-based fermented foods [235].

In cereal-based fermentations, yeast activity contributes to both the nutritional enhancement and sensory modification of the substrate. For example, *S. cerevisiae* used in the fermentation of multigrain products can improve not only the nutritional profile (e.g., increased protein and fiber) but also the texture and palatability of the final product by altering starch structure and reducing antinutrients that may affect taste [236]. The increase in total phenolic and flavonoid contents during fermentation enhances antioxidant potential, which may also influence the bitterness or astringency perception in the food matrix. Furthermore, the enzymatic degradation of phytates by *S. cerevisiae* var. *boulardii* releases minerals that can alter mouthfeel, while the metabolic byproducts of yeast activity, such as esters and alcohols, contribute to the aroma and flavor profile, improving the sensory appeal of the product [237].

In addition to nutritional and functional benefits, *S. cerevisiae* var. *boulardii* exhibits antimicrobial properties that can improve food safety without adversely affecting sensory quality. Its ability to degrade mycotoxins (e.g., aflatoxins, patulin) can reduce off-flavors or bitterness associated with microbial spoilage or contamination [238]. Thus, the inclusion of probiotic yeasts in food processing not only enhances microbial stability but also preserves or improves sensory attributes critical to consumer acceptance.

Consumer acceptance of probiotic-enriched foods is also influenced by a complex interplay of demographic, cultural, and psychographic factors [239]. Age and health status often shape perceptions, with younger consumers and those with higher health consciousness demonstrating greater willingness to purchase functional foods, whereas older populations may value probiotic benefits primarily in the context of disease prevention or management [240,241]. Gender differences have also been observed, with women generally exhibiting stronger interest in probiotic products, possibly due to greater health awareness and engagement with nutritional information [242]. Educational level and socioeconomic status can further impact acceptance, as individuals with higher education are typically more aware of probiotic functionality, while affordability remains a barrier for lower-income groups [242,243]. Cultural background plays a decisive role, as populations with a long-standing tradition of fermented foods, such as in parts of Asia and Eastern Europe, tend to accept probiotic fortification more readily than consumers in regions where probiotics are primarily introduced through supplements or fortified dairy products. Additionally, sensory attributes, convenience, and trust in labeling significantly affect purchase intent, with negative changes in taste or texture often outweighing perceived health benefits [244,245,246,247]. These findings underscore the importance of tailoring probiotic food development and marketing strategies to specific consumer segments, considering both cultural dietary norms and demographic profiles to optimize acceptance.

## 7. Regulatory Considerations for Probiotic Food Products

The regulatory landscape for probiotic food products is complex and heterogeneous, varying significantly across global jurisdictions. Probiotic-containing foods are subject to specific requirements concerning microbial strain identity, safety, functional claims, labeling, and product stability. These regulations are intended to ensure consumer safety and accurate communication of the health benefits associated with probiotics. However, the absence of harmonized global standards presents challenges for the development, marketing, and international trade of probiotic food products.

There is no universally accepted legal definition of probiotics, although the working definition established by the Food and Agriculture Organization (FAO) and World Health Organization (WHO) as “live microorganisms which when administered in adequate amounts confer a health benefit on the host” [1] is widely referenced in scientific and regulatory discussions. The International Scientific Association for Probiotics and Prebiotics [2] suggested minor corrections and defined probiotics as “live microorganisms that, when administered in adequate amounts, confer a health benefit on the host”. Jurisdictions differ in their classification of probiotic products. In many regions, probiotics added to foods are regulated either as foods, FFs, or dietary supplements, depending on their formulation and intended use.

For example, in the United States, the Food and Drug Administration (FDA) does not recognize probiotics as a distinct regulatory category but assesses them within the existing frameworks applicable to conventional foods, dietary supplements, or food additives. Probiotic strains intended for human consumption must either be designated as Generally Recognized as Safe (GRAS) or be approved through the food additive petition process, depending on their intended use and the available safety data.

The FDA allows the use of probiotics in food and dietary supplements under varying regulatory pathways, depending on the type of claim associated with the product. Three primary categories can be distinguished:

(i). Probiotics in food or supplements without health claims,
(ii). Probiotics in food or supplements with specific health claims, and
(iii). Probiotics classified as drugs.

For probiotics marketed without any explicit health claims, the strains must belong to species with a recognized safety profile and a documented history of safe use (FDA). These products are typically regulated as foods or dietary supplements and are not subject to pre-market approval, provided they are labeled truthfully and do not mislead consumers.

In contrast, the European Union (EU) employs a more structured approach under the oversight of the European Food Safety Authority (EFSA). One foundational element of the EFSA safety assessment process is the Qualified Presumption of Safety (QPS) framework. The QPS concept streamlines the safety evaluation of microorganisms intentionally added to food and feed by pre-establishing safety credentials for taxonomically defined groups of organisms. To be granted QPS status, a microorganism must meet four critical criteria:

(i). Taxonomic identity must be clearly and unequivocally defined;
(ii). The existing body of scientific knowledge must be sufficient to support a comprehensive safety assessment;
(iii). The absence of pathogenic properties must be demonstrated and substantiated; and
(iv). The intended use must be well characterized and appropriate [11].

In addition to microbial safety evaluation, the use of probiotics in EU food products is regulated by Regulation (EC) No 1924/2006 [248] on nutrition and health claims made on foods. This regulation provides a legal framework that mandates all claims to be scientifically substantiated, not misleading, and easily understood by the average consumer. Notably, under this regulation, the term “probiotic” is often interpreted as an implied health claim and, as such, may not be used in labeling or marketing unless it is accompanied by a health claim that has been officially authorized following EFSA evaluation. As for the latest assessments, very few probiotic-related health claims have received approval, primarily due to insufficient strain-specific evidence demonstrating a clear cause-and-effect relationship between the probiotic and the claimed health benefit.

### 7.1. Microbial Strain Identification and Characterization

Accurate identification and characterization of probiotic strains is a fundamental prerequisite for their use in food and therapeutic applications, as strain-specific properties critically influence both safety and efficacy. Probiotic functionality is not universally conserved across species or even subspecies; therefore, characterization at the strain level is required to ensure reproducibility of health effects and compliance with regulatory and scientific standards.

Initial taxonomic classification involves phenotypic, biochemical, and genotypic assessments. While traditional methods such as Gram staining, catalase testing, and carbohydrate fermentation profiles (API or Biolog systems) provide preliminary identification, they lack resolution at the strain level. Genotypic methods are considered the gold standard for precise taxonomic assignments. Methods such as PCR-based methodologies (DGGE/TGGE, PCR-RFLPS, qRT-PCR, WGS, metagenomics, etc.) and non-PCR-based methodologies (Maldi-TOF, Microarrays, PFGE, RFLPs) are widely used for strain identification. In Grujović et al. [4] a plethora of molecular tools and corresponding features for identification are presented in detail.

Following comprehensive identification, detailed strain-specific in vitro and in vivo characterization is essential to assess the suitability of a candidate probiotic for food or therapeutic use. As previously outlined, this includes the evaluation of key functional attributes, such as survival under gastrointestinal conditions (acid and bile tolerance), adhesion to intestinal epithelial cells, antimicrobial activity against pathogens, and relevant metabolic functions (e.g., short-chain fatty acid production, enzyme activities) [249]. In parallel, a rigorous safety assessment must be conducted, encompassing antibiotic resistance profiling according to EFSA breakpoints, hemolytic potential, the presence of virulence or toxin genes, and the capacity to produce biogenic amines. Furthermore, stability and technological properties, including tolerance to thermal, oxidative, osmotic, and desiccation stress, should be investigated to ensure viability during food processing, storage, and distribution.

For regulatory approval and reproducibility, each strain must be deposited in an internationally recognized culture collection (e.g., ATCC, DSMZ) under a unique accession number. Comprehensive documentation, including genomic sequences, phenotypic characteristics, and safety data, is crucial to ensure traceability, facilitate regulatory compliance, and support scientific transparency and reproducibility.

### 7.2. Labeling Requirements

To ensure accurate identification and effective functionality of probiotics in food products, it is recommended that the microbial species (single or multiple strains of live microorganisms) be clearly stated on the product label. When strain-level selection has been applied, critical due to the strain-specific nature of probiotic effects, the specific strain designation should also be included [250,251]. Accurate enumeration of viable probiotic microorganisms is essential, and labels should declare the viable counts of each strain at the end of the product’s shelf life to guarantee efficacy [250]. While products typically range from 1 to 10 billion CFU per dose, higher counts do not necessarily confer greater benefits [251]. Consumer safety is ensured through the use of well-characterized strains with a documented history of safe use, rigorous control of processing and storage conditions, and adherence to regulatory standards. Strain identity and functional attributes must be validated, and microbial activity must be maintained throughout food processing, handling, and storage [250]. Robust quality assurance systems, including Good Manufacturing Practices (GMP) [252] and implementation of Codex General Principles of Food Hygiene and HACCP-based food safety systems [253], minimize risks of contamination, spoilage, or unintended microbial proliferation. Accurate CFU enumeration, combined with validated safety protocols, ensures that probiotics are delivered in a form that is both efficacious and safe for human consumption [251]. Collectively, these practices support regulatory compliance and foster consumer trust in the safety and effectiveness of probiotic foods.

## 8. Consumer Perception and Market Trends Related to Probiotic Foods

Consumer perception plays a pivotal role in the acceptance and success of probiotic-enriched FF. Increasing public awareness of the link between diet, gut health, and overall well-being has driven a growing demand for foods that offer health-promoting benefits beyond basic nutrition [254]. Probiotics, in particular, are perceived positively due to their association with digestive health, immune modulation, and disease prevention. This favorable perception is reinforced by marketing strategies emphasizing “natural” health benefits, which resonate with health-conscious consumers seeking preventive healthcare solutions through diet.

Market analyses indicate a consistent upward trajectory in the global probiotic food sector. According to recent industry reports, the global probiotic food and beverage market is projected to grow substantially, driven by increasing health awareness, an aging population, and rising incidences of lifestyle-related disorders such as obesity, metabolic syndrome, and gastrointestinal diseases. Dairy-based products, including yogurt and fermented milk beverages, continue to dominate the market due to their traditional acceptance and well-established probiotic efficacy [255]. However, there is a growing trend toward non-dairy alternatives, including plant-based beverages, fermented vegetables, cereals, and functional beverages, in response to rising veganism, lactose intolerance, and allergy concerns [256].

Table 5 presents an overview of commercially available probiotic products and their health benefits. In the non-dairy sector, fruit and vegetable juices have proven to be effective carriers for probiotics due to their nutrient-rich composition and consumer appeal. For example, GoodBelly^®^ Probiotic Juice (USA), fortified with *Lpb. plantarum* 299v, is marketed for digestive health and is suitable for lactose-intolerant individuals [257]. Similarly, plant-based probiotic beverages, such as Yakult^®^ 1000 in Japan, utilize *Lcb. casei* Shirota to promote gut health and immune function [258]. Probiotic incorporation into cereal-based products, such as Attune^®^ Probiotic Chocolate Bars (USA) containing *B. lactis* HN019 and *Lb. acidophilus* NCFM, demonstrates the viability of shelf-stable delivery systems [259]. Innovations in snack and protein-rich products highlight the expanding scope of probiotic applications. Shelf-stable fruit snacks, such as Barnana^®^ Probiotic Banana Bites (USA) containing *Bacillus coagulans* GBI-30, 6086, offer heat-stable probiotics with convenience for active consumers [260]. In the meat sector, Salgot^®^ Fuet Probiotic Sausage (Spain) incorporates *Lpb. plantarum* (as a starter) and *Lb. acidophilus* (probiotic microorganism) to improve safety through pathogen inhibition while enhancing sensory qualities [261].

Despite the promising outlook, consumer understanding of probiotics remains limited and often influenced by inconsistent labeling and unsubstantiated health claims. Surveys have shown that many consumers are unaware of strain specificity, required dosages, and the importance of viability at the point of consumption. Consequently, trust in probiotic products is influenced not only by scientific evidence but also by brand reputation, product transparency, and third-party endorsements [266,267]. Educational initiatives and clearer regulatory guidelines are needed to bridge the knowledge gap and enhance informed decision-making among consumers.

## 9. Future Research Directions for Improving Probiotic Efficacy in Functional Foods

Future research aimed at enhancing the efficacy of probiotics in FFs must address several key challenges spanning microbial characterization, delivery systems, host interactions, and regulatory frameworks. A fundamental priority is the detailed, strain-specific characterization of probiotic organisms. Advances in omics technologies, including genomics, transcriptomics, proteomics, and metabolomics, can facilitate the identification of functional traits such as acid and bile tolerance, mucosal adhesion, antimicrobial activity, and immunomodulatory potential [249]. These insights will enable the selection of strains with defined health benefits and robust performance in food matrices.

Another critical area of development is the optimization of microencapsulation and delivery systems. Encapsulation using biopolymers, nanoemulsions, or multilayer coatings offers protection against environmental stresses encountered during food processing and gastrointestinal transit [193]. Traditional techniques such as spray drying and extrusion remain widely used due to their cost-effectiveness and ease of implementation. However, they often fall short in protecting probiotic viability under harsh processing and storage conditions. In contrast, novel encapsulation methods, particularly nanoencapsulation and smart delivery systems, offer superior protection and targeted release, although they present challenges in terms of complexity, cost, and regulatory approval [201,202,206]. Future studies should explore targeted delivery strategies that enable the release of probiotics at specific sites within the gastrointestinal tract, thus enhancing colonization and functional activity.

Equally important is the need for comprehensive investigations into matrix–strain compatibility. Food matrices significantly influence probiotic viability and activity, and the interactions between specific strains and components of dairy, plant-based, meat, or cereal-based foods require systematic analysis [268]. Such research will guide the formulation of products that maintain probiotic stability without compromising sensory quality or nutritional value.

Understanding the mechanisms by which probiotics confer health benefits remains a research priority. Clinical studies complemented by in vitro and in vivo models are necessary to elucidate host–microbe interactions, microbiota modulation, and the downstream effects on metabolic and immune pathways [269,270]. These insights are particularly relevant for the development of personalized probiotic interventions tailored to individual microbiome profiles and health needs.

The integration of probiotics into personalized nutrition frameworks represents a promising frontier. By aligning probiotic formulations with individual dietary patterns, microbiota compositions, and health statuses, personalized interventions may enhance efficacy and clinical outcomes [271]. Advances in bioinformatics and microbiome analytics will be instrumental in this regard.

Regulatory harmonization is also essential to ensure product quality and consumer confidence. There is a pressing need for standardized criteria for probiotic strain identification, efficacy evaluation, and health claim substantiation [251,272]. Harmonized guidelines across jurisdictions will facilitate innovation and streamline market approval processes.

Lastly, ensuring the long-term stability of probiotic products remains a technical challenge [273]. Research should focus on modeling probiotic viability under different storage conditions and packaging systems to ensure label-claimed efficacy throughout the product’s shelf life. Moreover, combining probiotics with synergistic components such as prebiotics, polyphenols, and antioxidants could further enhance survival and functional efficacy [274]. Investigating these combinations using multi-omics approaches will provide a deeper understanding of their interactive mechanisms and potential health benefits. Collectively, these research directions will contribute to the development of next-generation probiotic-enriched FFs that are effective, safe, and widely accepted by consumers.

## 10. Conclusions

The incorporation of probiotics into food products represents a practical, consumer-friendly strategy for delivering health-promoting microorganisms as part of the daily diet. This approach not only supports long-term adherence to probiotic intake but also facilitates sustained health benefits through routine consumption. However, the efficacy of probiotic-enriched foods is highly dependent on several critical factors, including the viability and functionality of probiotic strains throughout food processing, storage, and gastrointestinal transit. Innovations in food technology, such as microencapsulation, co-formulation with prebiotics, and controlled fermentation techniques, play a pivotal role in preserving probiotic stability and ensuring their effective delivery.

Despite growing consumer interest and scientific support for probiotic foods, challenges remain in the optimization of strain selection, dosage, and matrix compatibility. Continued research, particularly focusing on strain-specific mechanisms, host–microbe interactions, and clinically validated outcomes, is essential for the development of targeted and efficacious probiotic formulations.

Regulatory frameworks for probiotic food products are comprehensive, requiring rigorous microbial identification, safety assessment, substantiation of health claims, and accurate labeling. These measures are necessary to safeguard public health and ensure product integrity. Nevertheless, inconsistencies and lack of harmonization among international regulatory systems pose significant barriers to innovation and market accessibility. Establishing unified, science-based global standards will be critical to fostering the development and acceptance of probiotic-containing FFs.

Finally, probiotic foods offer a palatable and effective vehicle for enhancing human health. Strategic advancements in strain characterization, technological innovation, clinical validation, regulatory alignment, and consumer perception of FF are essential to fully realize the potential of probiotics in FF applications.

## Figures and Tables

**Table 1:** Summary of probiotic strains, health, and functional properties.

Probiotic Strain(s)	Benefit and Outcome	Reference
*Lactobacillus johnsonii* La1, *Ligilactobacillus salivarius* (formerly classified as *Lactobacillus salivarius*), *Lb. acidophilus* LB, *Lactiplantibacillus plantarum*, *Lcb. rhamnosus*	Improve lactose tolerance and digestion through inhibition of pathogen growth and reduced urease activity, aiding survival in acidic stomach environments	[24,27]
*Lcb. rhamnosus* GG, GR-1, LC-705, *Lcb. casei* (incl. Shirota), *Lb. acidophilus* (incl. HN017, LA-2), *Lmb. reuteri* RC-14, *Levilactobacillus brevis*	Support microbiota homeostasis; improve intestinal function and immune response; prevent/treat diarrhea and genitourinary infections; modulate gut microbiota and inflammation in IBS; aid in colon cancer prevention; restore vaginal microbiota	[23,24,28]
*Lcb. rhamnosus* MG4502, *Lactobacillus gasseri MG4524*, *Lmb. reuteri MG5149*, *Weissella cibaria MG5285*	Exhibit antioxidant activity through DPPH and ABTS radical scavenging assays and α-glucosidase inhibitory activities; promote anti-adipogenesis by reducing lipid accumulation	[29]
*Lactobacillus* spp. (general)	Control *Helicobacter pylori* colonization; aid in cholesterol reduction and B-vitamin synthesis	[30]
*Enterococcus faecium*	Inhibit the growth of pathogens such as *Listeria innocua*, *Micrococcus luteus*, *Escherichia coli*	[31]
*S. thermophilus*	Improve intestinal microbial balance and function, especially in combination with other probiotics; immune stimulation (cytokine release)	[24,32]
*Bifidobacterium* spp. (including *B. bifidum*, *B. lactis* BB-12, HN019, *B. animalis* subsp. *lactis*)	Support gut microbial balance; prevent/treat infectious diarrhea; aid in cholesterol reduction and B-vitamin synthesis; contribute to immune response; help prevent allergic symptoms and colon cancer	[24,33]
*Propionibacterium* spp. (*P. freudenreichii*)	Contribute to colon cancer prevention when combined with other probiotic strains	[24,34]
*S. boulardii*	Inhibit growth of harmful intestinal bacteria, supporting gut health	[35,36]
*S. cerevisiae*	Provide antigenotoxic and cytotoxic effects; enhances gut health, flavor, and probiotic function	[37]
*S. cerevisiae*, *S. boulardii*	Enhance dairy production efficiency, milk yield, and quality; improve nutrient digestibility and microbial protein synthesis; normalize gut flora; treat gastrointestinal disorders	[38,39]
*Kluyveromyces lactis*, *K. marxianus*	Produce beneficial dairy enzymes; improve dairy product performance, texture, flavor, and probiotic qualities; improve human fecal microbiota composition	[40,41]
*Debaryomyces hansenii*	Produce aromatic compounds that enhance flavor and reduce fermentation time in cheese production; shows probiotic effects in animal models; possesses antioxidant, antimicrobial, and immune-modulating properties	[42,43]
*Yarrowia lipolytica*	Produce enzymes for fat breakdown, enhancing texture and taste	[44]
*Candida milleri*	Promote digestive health and enhance the immune system by fostering a balanced gut microbiome	[26]
*Candida stellimalicola*, *Cyberlindnera (Pichia) jadinii*	Improve lactate degradation during fermentation in dairy products (Dadih)	[45]
*Pichia kudriavzevii* (esp. strain 5S5)	Show strong probiotic traits, like gastrointestinal tract (GIT) survival, adhesion, hydrophobicity	[46]
*Pichia kluyveri* LAR001	Tolerate low pH (2.5); survives gastrointestinal (GI) conditions; exhibits antimicrobial activity	[47]
*Hanseniaspora uvarum* PIT001	Exhibit probiotic potential under GI stress conditions	[47]
*Candida intermedia* ERQ001	Exhibit probiotic potential under various fermentation conditions	[47]

**Table 2:** Overview of clinical studies highlighting the health benefits of probiotics delivered through food.

Probiotic	Dosage	Duration of Study	Participants	Health Benefit	Reference
*Lcb. casei* Shirota	Probiotic drink with live *Lcb. casei* Shirota 6.5 × 10^9^	21 days	164 spinal cord injury (SCI) patients	Reduction of antibiotic-associated diarrhea in hospitalized SCI patients	[71]
Yakult (*Lcb. casei* Shirota)	1 bottle of fermented milk per day; 1 × 10^11^ CFU per bottle	8 weeks	23 normally nourished students, aged 23 years	Alleviation of stress-induced intestinal dysfunction symptoms	[72]
*Lcb. rhamnosus* CGMCC 1.3724	Probiotic and peanut oral immunotherapy (PPOIT)	2 to 5 weeks	64 children (1–10 years) with peanut allergy	Effective in inducing possible sustained immune changes that suggest modulation of the peanut-specific immune response	[73]
*Lb. acidophilus* L1	200 g of yogurt with *Lb. acidophilus* L1	10 weeks	48 volunteers with blood cholesterol concentrations ranging from 5.40 to 8.32 mmol/L	2.4% reduction in cholesterol levels compared to the placebo group	[74]
*Limosilactobacillus fermentum* ME-3 (formerly classiefied as *Lactobacillus fermentum*)	Fermented goat milk, unknown daily consumption amount	3 weeks	21 volunteers	Extended protection period against lipoprotein oxidation, and reduced levels of oxidized LDL, peroxidized lipoproteins, 8-isoprostane, and improved glutathione redox ratio	[75]
	Fermented milk (6.3 × 10^11^ CFU daily); capsules (1.6 × 10^9^ CFU daily)	3 weeks	21 volunteers for fermented milk; 24 volunteers for capsules	Improved total antioxidant activity and antioxidant status in blood: 6% and 9% for fermented milk, and 4% and 2.5% for capsules	[76]
*S. thermophilus*	Daily intake of milk fermented with *S. thermophilus* (test group) and a placebo group (milk without the bacterium)	12 weeks	30 individuals with an average LDL cholesterol level of 140 mg/dL	Proven benefit for healthy individuals and patients with mild hyper-LDL-cholesterolemia	[77]
*Bifidobacterium lactis* HN019	Daily intake of 80 mL of milk fermented with 2.72 × 10^10^ CFU of *B. lactis* HN019	45 days	51 patients with metabolic syndrome	7.7% reduction in cholesterol levels; 13% reduction in LDL cholesterol	[78]

**Table 3:** Pros and cons of different fermentation techniques.

Fermentation Method	Pros	Cons	Reference
Traditional spontaneous fermentation	Natural inoculation with diverse indigenous strains; enhances flavor complexity and cultural authenticity while remaining low-cost and requiring minimal equipment	Uncontrolled microbial composition poses safety risks; variability between batches and unpredictable probiotic levels may occur due to the lack of process control; potential safety risk due to pathogens	[4,115]
Controlled batch fermentation	High reproducibility and safety under defined conditions; optimized pH, temperature, and oxygen control improve probiotic viability and yield, enabling industrial-scale production and regulatory compliance	Requires advanced control systems and higher operational costs; often limited to monocultures or defined consortia, reducing microbial diversity	[84,116,117]
Immobilized cell fermentation	Enhanced probiotic stability and reusability; immobilization improves survival during processing and gastrointestinal transit, supports continuous fermentation, and may reduce long-term costs	Complex immobilization processes and scale-up challenges; mass transfer limitations and the cost of carriers can hinder industrial application	[98,117]
Co-fermentation techniques	Synergistic microbial interactions boost probiotic performance; enhance vitamins, peptide, and bioactive production while improving texture and flavor profiles	Strain compatibility must be carefully managed; antagonism, competition, and the complexity of monitoring microbial dynamics can be challenging	[5,99,118]
Synbiotic fermentation	Combines probiotics and prebiotics for synergistic effects; prebiotics improve probiotic survival and colonization, enhance nutrient bioavailability, inhibit spoilage organisms, and improve texture, especially in dairy and plant-based foods	Effectiveness depends on correct pairing of probiotics and prebiotics; formulation must be optimized to avoid loss of viability, increased costs, off-flavors, or undesirable texture changes; health claims require scientific validation	[103,110,115,119]

**Table 4:** Key features, advantages, challenges, and examples of different matrices for probiotic delivery.

	Matrix	Key Features	Probiotic Strains	Prebiotics	Advantages	Challenges	References
Dairy-Based	Fermented Skim Milk	Prepared from skim milk powder with added inulin	*Lb. bulgaricus*, *Lb. acidophilus*, *Lcb. rhamnosus*, *B. lactis*	Inulin	Improved viability of specific probiotic strains	Strain-specific responses and storage time significantly influence probiotic viability and product firmness	[144]
	Yogurt	Semi-solid fermented milk; buffering capacity	*Lactobacillus* *helveticus*, *Lcb. casei*, *B. animalis*	Polydextrose, Lactitol	Good probiotic survival; favorable sensory properties	Viability may decrease over prolonged storage	[145,146,147]
	Cheese	Semi-hard/hard, low moisture, neutral pH	*Lcb. paracasei*, *Lpb. plantarum*, *Lb. delbrueckii*	FOS—1%, Inulin, Maltodextrin	Long shelf life; protects probiotics from acidity	Flavor changes if probiotic metabolism is excessive	[148,149,150]
	Fermented Beverages (kombucha and kefir)	Liquid form; easily consumed; often fruit-flavored; health benefits	*Lb. acidophilus*, *Lcb. casei*, *Lcb. rhamnosus*, *B. lactis*, *Bacillus coagulans* and yeasts *Km. marxianus*, *S. cerevisiae*, *S. boulardii*	Sucrose, bee pollen, honey	Suitable for pediatrics and elderly; enhanced survival with stabilizers or encapsulation	Higher oxygen exposure; shear stress during processing	[151]
	Infant Formula	Lyophilized probiotics and varying GOS levels were added to a milk-based formula; nutritional and mineral bioavailability assessments were conducted using a rat model	*B. bifidum*, *B. longum*	GOS—1.2%, 5% and 10%	Synbiotic formulas improved mineral bioavailability, particularly in younger rats	Lack of probiotic viability data at consumption point	[152]
	Ice Cream/Frozen Desserts	Frozen dairy products; low acidity	*Lcb. rhamnosus*, *Lcb. casei*, *Lb. acidophilus*	Inulin	Cryoprotectants aid survival; appealing to children	Freezing stress; viability loss over time	[153,154]
Meat and egg-Based	Fermented Meat	Rich in protein, fat; fermentation promotes safety	*B. longum*, *Enterococcus faecium*, *Lcb. casei Lcb. paracasei*, *Lcb. rhamnosus*, *Lpb. plantarum Lb. acidophilus*, *Llp. sakei*, *Lmb. fermentum*, *Pediococcus acidilactici*	/	Improved flavor, safety; viable probiotics post-ripening	Salt and curing agents inhibit survival	[55]
	Cooked/Ready-to-Eat Meat	Heat processing; requires protection of probiotics	*Lactobacillus*, *Bifidobacterium*, *Enterococcus*, and *Pediococcus* genera	/	Convenient format; high protein content	Heat sensitivity; need encapsulation technologies	[155]
	Dry-Cured Meat	Long curing, low moisture	*Llb. curvatus*, *Llb. sakei*	0.2% organic sunflower honey or without prebiotics	Maintains viability over long shelf-life	Moisture reduction limits probiotic metabolism	[143]
	Spray-Dried Egg Powder	High protein, low moisture	*Lb. acidophilus*	/	Stability during drying and storage	Limited commercial applications; heat during drying	[156]
	Liquid Egg Products	Emulsifying, foaming properties	*Lcb. casei*, *Lgb. salivarius*	Different fruit juices	Functional stabilization of probiotics	Thermal processing can reduce viability	[157]
	Egg-Based Dressings	Fat-rich, acidic	*Lcb. paracasei* subsp. *paracasei*	Inulin	Protection from acidity and fat matrix	Sensory challenges; antimicrobial egg components	[158]
Plant-Based	Cereal-Based	High in carbohydrates, β-glucans, dietary fiber	*B. lactis*, *Lpb. plantarum*, *Lb. acidophilus*, *Lcb. casei*, *S. boulardii*, *B. coagulans*, *B. subtilis*	/	Natural prebiotics; good texture and viscosity; supports probiotic growth	Low in essential amino acids; may need supplementation	[159]
	Legume-Based	High in protein, oligosaccharides, minerals	*Lb. delbrueckii* ssp. *bulgaricus*, *Lb. acidophilus*, *Lcb. casei*, *Leuc. mesenteroides*, *Lpb. plantarum subsp. plantarum*, *Lcb. rhamnosus*	/	Good buffering capacity; high nutritional value; stable viability	Beany flavor; requires flavor masking or enzymatic treatment	[160]
	Fruit Juices	High in sugars, polyphenols, vitamins, antioxidants	*Lpb. plantarum*, *Lb. acidophilus*, *Lcb. casei*, *Lcb. rhamnosus*	FOS, GOS	Consumer-friendly flavor; antioxidant synergy; no need for added sugar	Low pH may reduce viability; phase separation	[134]
	Vegetable Juices	Moderate sugar, high fiber, minerals, polyphenols	*Lb. acidophilus*, *Lpb. plantarum*, *Lcb. rhamnosus*, *Lmb. reuteri*, *B. lactis*	/	Nutrient-rich; supports functional benefits (e.g., antioxidants)	Astringent taste, color, and flavor may reduce acceptance	[161]
	Nut-Based Beverages	Rich in fats (especially MUFA/PUFA), moderate protein, low sugar, no lactose	*B. animalis*, *Lb. acidophilus*, *Lpb. plantarum*	/	Appealing for vegan/clean-label products; healthy fats; pH around 6.5	Moderate protein; emulsification and separation issues	[162]
	Plant-based Beverages	High in omega-3 fatty acids, fiber, and phytochemicals, low protein	*Lactobacillus* and *Bifidobacterium* genera	/	Nutrient-dense; potential prebiotic effect, rich in vitamins	High microbial contamination, short shelf life, functional efficacy of probiotics was not investigated, stability concerns, flavor and texture inconsistencies, low nutritional value	[163]
Bakery products	Bread, Cookies, and Biscuits	Rich in carbohydrates, proteins, dietary fibers, nutritionally valuable food matrices, wide consumer acceptability	Encapsulated *Lcb. rhamnosus* and *Lpb. plantarum*	/	Non-dairy alternatives, catering to individuals with lactose intolerance or dairy allergies	High baking temperature, which can significantly reduce probiotic viability, probiotic stability during processing, storage, and gastrointestinal passage remains complex	[138,139]
Confectionery and Snacks	Chocolate	Hight fat content	*Leuc. mesenteroides*	Flax seeds	Probiotic survived throughout the storage period, with antioxidant activity being retained in the product	Not defined	[140]
	Candy	High potential to benefit consumer health	*Lpb. plantarum*	Polysaccharide extracts from three different Indian seaweeds, inulin	High probiotic surveillance	Unknown influence on sensory acceptance	[164]
	Mousse	Improved probiotic survival to in vitro gastrointestinal stress	*Lb. acidophilus*	Inulin, FOS	Probiotics and prebiotics proportion was kept constant	Not defined	[165]

FOS—fructooligosaccharides; GOS—galactooligosaccharides; */*—the absence of a prebiotic.

**Table 5:** Overview of commercially available probiotic products and their health benefits.

Commercial Product	Probiotic Strain(s)	Delivery Format/Technology	Claimed/Reported Benefits	Reference
Activia^®^ Yogurt	*B. lactis* DN-173 010/CNCM I-2494	Direct inoculation into milk base	Supports gut transit and microbiota balance	[262]
Lifeway^®^ Kefir	*S. diacetylactis*, *Lb. lactis*, *Lb. rhamnosus*, *Lb. acidophilus*, *Lb. reuteri*, *Lpb. plantarum*, *Lcb. casei*, *Saccharomyces florentinus*, *Leuconostoc cremoris*, *B. longum*, *B. breve*, *B. lactis*	Mixed culture, fermented milk drink	Supports immunity, digestion, and microbiota diversity	[263]
SVELTY^®^ Gastro Protect	*Lb. johnsonii* La1	Fermented milk drink	Control *H. pylori* infection and stomach discomfort	[259]
Yakult^®^ 1000	*Lcb. casei* Shirota	Fermented skim milk base and soybean with high probiotic count	Improves gut health; supports immune function; increases beneficial bacteria; reduces the harmful bacteria	[258]
Yakult	*B. brevis*, GOS		Prevent dryness of the skin; beneficial effects on the intestinal conditions; stimulation of defecation; decrease in phenol production by gut bacteria	[264]
GoodBelly^®^ Probiotic Juice	*Lpb. plantarum* 299v, *B. lactis* Bi-07, *Lb. acidophilus*	Refrigerated juice, direct inoculation	Supports digestive health; lactose-free; stimulates the immune system; synthesis of vitamins (vitamin B and vitamin K); absorption of key nutrients (calcium, magnesium, and iron)	[257]
PERKii^®^	*B. lactis*, *Lcb. casei*	Natural fruit juices with no artificial colors, flavors, or sweeteners	Supports gastrointestinal health and immune system	[259]
Attune^®^ Probiotic Chocolate Bars	*Lb. acidophilus*, *Lcb. casei*, *B. lactis*	Dark chocolate coating as protective matrix; organic brown rice crisps	Supports digestive health; shelf-stable	[259]
Probiotic Banana Bites	*Bacillus coagulans*	Spore-form probiotics in dried banana pieces	Digestive health; stable at ambient temperature	[260]
Salgot^®^ Fuet Probiotic Sausage	*Lpb. plantarum* (as a starter), *Lb. acidophilus* (as a probiotic)	Starter culture in dry-fermented sausage	Pathogen inhibition; improved flavor profile	[161]
	*Lcb. rhamnosus* HN001	Probiotic culture	Improved inflammatory and immunological markers (CRP and TNFα); improved antioxidant plasmatic markers and butyrate production	[265]

## Data Availability

No new data were created or analyzed in this study. Data sharing is not applicable to this article.

## Abbreviations

The following abbreviations are used in this manuscript:

LAB	Lactic Acid Bacteria
GRAS	Generally Regarded as Safe
QPS	Qualified Presumption of Safety
FFs	Functional Foods
GI	Gastrointestinal
GIT	Gastrointestinal Tract
SCOBY	Symbiotic Culture of Bacteria and Yeast
SDGs	Sustainable Development Goals
SCI	Spinal Cord Injury
PPOIT	Probiotic and Peanut Oral Immunotherapy
FOSs	Fructooligosaccharides
GOSs	Galactooligosaccharides
CLA	Conjugated Linoleic Acid
A_w_	Water Activity
EPSs	Exopolysaccharides
EFSA	European Food Safety Authority
GPP	Grape Pomace Pectin
